# Personal beliefs versus evidence-based decisions: vaccination behavior and doubts about antidepressants of students of medicine are affected by conspiracy theories

**DOI:** 10.1192/j.eurpsy.2023.2388

**Published:** 2023-07-19

**Authors:** V. Pisl, D. Kestlerova, J. Losak, T. Skorkovsky, J. Vevera

**Affiliations:** 1 Psychiatry clinic, Faculty of Medicine in Pilsen, Charles University, Plzen, Czech Republic

## Abstract

**Introduction:**

When medical professionals are obliged to adhere to *lege artis* and evidence-based decision-making, they need to interpret available evidence. As a complex cognitive process, however, such interpretation may be affected by socio-cognitive biases and predispositions. For instance, the conspiracy mentality (the general readiness to believe in conspiracy theories) or biological determinism (the belief that human lives are determined biologically) affect attitudes to antidepressant medication and vaccination in the public. Little is known about the effects of these variables on the decision-making of clinicians or students of medicine.

**Objectives:**

The study examines the effects of conspiracy mentality (CM), covid-related conspiracy beliefs (CCBs), and biological determinism (BD) on the doubts students of medicine have about antidepressants and on their uptake of the booster dose of COVID-19 vaccine.

**Methods:**

CM, CCBs and BD were measured in May 2022 in a sample of 179 students of medicine (115 females), using the Conspiracy Mentality Questionnaire by Bruder et al. (2013), set of items measuring CCBs developed by Imhoff and Laberty (2021), and the Biological Basis scale by Bastian and Haslam (2006), respectively. The doubts about antidepressants were measured by the Antidepressant Conspiracy Scale by Natoli et al. (2021) tapping participants’ beliefs that drugs and antidepressants specifically are ineffective and promoted and prescribed for financial gains. Logistic and linear regression models were used to predict respondents’ vaccine uptake and doubts about antidepressants.

**Results:**

Booster vaccine uptake was predicted by BD (OR = 1.45; *p* < .05) and CCBs (OR = .73*; p* < .05), together explaining 7% of the variance. Booster vaccine uptake was not predicted by CM.

Doubts about antidepressant medication were predicted by CM (*b* = .17, *p* < .001) but not BD (*p* = .89), together explaining 10% of the variance.

**Conclusions:**

The doubts students of medicine have about antidepressants and their vaccination behavior was predicted by their biological determinism, belief in conspiracy theories and general conspiracy mentality. Although the relationships were weak, they support claims that health-related beliefs and behaviors of students of medicine are related to their implicit beliefs and socio-cognitive predispositions. These personal factors may therefore affect their professional decision-making and should be addressed in medical education.

**Disclosure of Interest:**

None Declared

